# Risk Factors of Patients With Diarrhea for Having *Clostridioides (Clostridium) difficile* Infection

**DOI:** 10.3389/fmicb.2022.840846

**Published:** 2022-03-11

**Authors:** Vanessa Lang, Katrin Gunka, Jan Rudolf Ortlepp, Ortrud Zimmermann, Uwe Groß

**Affiliations:** ^1^Institute of Medical Microbiology, University Medical Center Göttingen, Göttingen, Germany; ^2^Asklepios Kliniken Schildautal Seesen, Seesen, Germany

**Keywords:** *Clostridioides difficile*, *Clostridium difficile*, risk factor, proton pump inhibitor, nosocomial infection, antibiotic, diuretics, torasemid

## Abstract

Nosocomial infections with *Clostridioides (Clostridium) difficile* have become an emergent health threat. We sought to define risk factors for a *C. difficile* infection (CDI) beyond the widely known ones, such as antibiotic use and prior hospital stay. We therefore focused on a group of patients with diarrhea in order to identify risk factors for *C. difficile* infection among this symptomatic cohort. A total of 121 hospitalized patients from Seesen/Germany with diarrhea were included who submitted a stool sample and were interviewed about their socio-demographic background, lifestyle and state of health using a standardized questionnaire. Antibiotic potential of diuretics was examined by agar diffusion test. *C. difficile* was identified in 29 patients resulting in a prevalence of 24.0%. The infection was hospital-acquired in most cases (*p* < 0.001, 82.1%; *n* = 23/28, versus 29/91, 31.9%). The generally accepted risk factor previous antibiotic use was confirmed in this study (*p* = 0.002, *n* = 23/28 CDI patients, 82.1%, versus *n* = 44/91 non-CDI patients, 48.4%). The following additional risk factors were identified: regular consumption of proton pump inhibitors; PPI (*p* = 0.011, *n* = 24/29, 82.8% vs. *n* = 52/92, 56.5%), CDI patients ate less vegetables (*p* = 0.001, *n* = 12/29, 41.4% vs. 69/92, 75.0%). The intake of the diuretic agent torasemid in patients with CDI (*p* = 0.005, *n* = 18/29, 62.1%) was higher than in patients without (*n* = 30/92, 32.6%). More patients with CDI had to undergo a surgery in the previous year (*p* = 0.022, *n* = 13/29, 44.8% vs. *n* = 21/92, 22.8%) and held more birds (*p* = 0.056, *n* = 4/29, 13.8%) than individuals of the negative group (*n* = 3/92, 3.3%). In conclusion, although no antibiotic potential was detected in diuretics, especially torasemid seems to have significant influence for the occurrence of a CDI as well as a nutrition poor in vegetables. A diet rich in vegetables represented a fourfold lower risk for a CDI (OR 0.240, CI (0.0720 - 0.796]).

## Introduction

*Clostridioides (Clostridium) difficile* was first described in 1935 in the intestinal microbiome of healthy neonates. Due to its difficulty of cultivation, it was initially termed *Bacillus difficilis* (Hall and O’Toole, 1935). Nearly forty years later, the relation between pseudomembranous colitis and *C. difficile* was revealed, especially after clindamycin treatment (Tedesco et al., 1974). During the last centuries *C. difficile* has been identified as the most common cause of antibiotic-associated diarrhea in industrialized countries. The most important virulence factors are the entero- and cytotoxins TcdA and TcdB as well as the binary toxin (CDT) that is expressed especially by hypervirulent strains. *C. difficile* infection (CDI) causes a variety of symptoms, ranging from mild diarrhea up to severe pseudomembranous colitis that can end up in bowel perforation (Kuijper and van Dissel, 2008; Leffler and Lamont, 2015). Our existing knowledge about potential risk factors of the living environment and nutritional habits of the patients seems not to be complete. This study aimed to explore the prevalence of *C. difficile* in inpatients of a rural community in Lower Saxony/Germany and the existence of further risk factors for CDI, focusing on current medication of the patients as well as the influence of lifestyle and living environment.

## Materials and Methods

### Study Design and Patients

This prospective study was approved by the Ethical Committee responsible for the participating hospital and the University Medical Center, Göttingen, Germany (29/3/11) and was performed during the period of September 2013 to April 2014 at the Asklepios Hospital Schildautal in Seesen, Germany. All inpatients with diarrhea anytime during hospitalization (three or more loose or liquid stools per day as defined by WHO) and of unknown origin at time of sampling were asked to participate and also provide a stool sample. All had to be at least 18 years of age and of sound mind to fill out the questionnaire about their socio-demographic background, living environment, and state of health during the last 12 months (see Supplementary data sheet 1). The sample size determination was calculated according to Kish’s formula (Kish, 1965) and resulted from an estimated prevalence range of 17-18% which was based on own epidemiological experience in this region of Germany (unpublished data). A case was defined as hospital-acquired when diarrhea occurred more than 48 h after hospital admission (Heister et al., 2019).

### Laboratory Methods

For this study on CDI risk factors, all stool samples from the study participants and animals eventually living in their household were cultured on respective *C. difficile* selective agar (bioMérieux, Marcy-l’Étoile, France) and incubated for 48 h under anaerobic conditions. MALDI-TOF mass spectrometry (Biotyper, Bruker Daltonics, Bremen, Germany) was subsequently used for identification of *C. difficile*. In addition, all stool samples were also tested for the presence of *C. difficile* toxin by using the Serazym^®^ C. difficile toxin A + B immunoassay (Seramun Diagnostica GmbH, Heidesee, Germany). If culture was positive and toxin test from stool was negative, toxin production was re-investigated directly from grown colonies. All stool samples from patients and animals were also tested for the presence of other pathogens by adequate laboratory methods.

Antibiotic potential of the diuretics used by the participants was examined using an agar diffusion test. Briefly, 100 μl of torasemid 10 mg, furosemide 40 mg and hydrochlorothiazide 25 mg tablets, separately diluted in 1 ml NaCl 0.9% solution, were spread on WHATMAN Grade AA 9 mm filter disks (GE HEALTHCARE, Chalfont St Giles, Buckinghamshire, United Kingdom). These filter disks were arranged onto Müller-Hinton agar plates (OXOID, Basingstoke, Hampshire, United Kingdom) containing spores of antibiotic-susceptible *Bacillus subtilis* (ATCC6633, LGC Standards GmbH Wesel, Germany). After 24 h of incubation at 37°C, the agar plates were analyzed for the presence of growth-inhibiting zones around the filter disks. For furosemide and torasemid, the test was also performed with 100 μl injection solution, 20 mg and 2 mg, respectively.

### Statistical Analysis

All data were entered in the IBM^®^ SPSS^®^ Statistics V24.0 program (IBM United States Software Announcement, Armonk, NY, United States) and Statistica (Statsoft GmbH, Hamburg, Germany) for statistical analysis. A first statistical overview was obtained by arranging items in frequency and cross tables. Significance testing was performed using Chi-square and Fisher’s exact test for nominal data, Mann-Whitney non-parametric test for data of an ordinal scale level and *t*-test for metric parameters. Predictors for the incidence of CDI were analyzed by the multivariate logistic regression (including criteria *p* < 0.2) and quoted by odds ratio. A *p*-value <0.05 was considered as statistically significant.

## Results

In this prospective study, a total of 121 inpatients with diarrhea during hospitalization were investigated. The patients had an average age of 70 years, consisting of nearly equal numbers of male (*n* = 64, 52.9%) and female (*n* = 57, 47.1%) individuals. All patients were of sound mind to give informed consent in accordance with the Declaration of Helsinki. *C. difficile* occurred with a prevalence of 24.0% (*n* = 29/121). The infection was hospital-acquired in most cases (*p* < 0.001, 82.1%; *n* = 23/28, versus 29/91, 31.9%). There was no indication for clustering of CDI patients.

### Individual Risk Factors for *Clostridioides (Clostridium) difficile* Infection

To identify putative risk factors for the exposition to the pathogen, *C. difficile*-positive as well as -negative diarrheic patients were compared with each other based on data provided in structured interviews (Supplementary data sheet 1 and Table 1). While there were no significant effects regarding the number of meals per day, the univariate analysis revealed that *C. difficile*-positive individuals ate significantly less vegetables (*p* = 0.001) and more instant food (*p* = 0.121) than others. According to the multivariate logistic regression model, a nutrition poor in vegetables was besides inpatient care the only factor showing significance (*p* = 0.020) for the risk of acquiring CDI (Table 2). With an odds ratio of 0.235, the risk for a CDI was nearly fourfold lower among those patients ranging vegetables among their daily nutrition. A significant effect was observed regarding the consumption of vegetables (*p* = 0.001); just 12 out of 29 (41.4%) *C. difficile*-positive patients consumed vegetables on a daily basis. In comparison, 69 out of 92 of the *C. difficile*-negative patient group (75.0%) ate vegetables every day (Table 1). Furthermore, patients with CDI also consumed more instant food (24.1%, *n* = 7/29) than patients without CDI (10.9%, *n* = 10/92, *p* = 0.121).

**TABLE 1:** Potential risk factors.

Univariate analysis of risk factors for CDI						
Factor	*C. difficile* positive		*C. difficile* negative		OR (95% CI)	p value
		
	%	(n/tested)	%	(n/tested)		
Male gender	62.1	18/29	50.0	46/92	1.636 (0.696-3.845)	0.256
Age (mean value in years)	73.5	29/29	68.7	92/92	-	0.121
Mean value of Body Mass Index (BMI) Missing observations	26.0	28/29 1	27.7	88/92 4	-	0.213
Surgery in previous 12 months	44.8	13/29	22.8	21/92	2.747 (1.141-6.616)	0.022*
Prior antibiotic medication in previous six months Missing observations	82.1	23/28 1	48.4	44/91 1	4.914 (1.718-14.054)	0.002*
Meropenem Missing observations	18.5	5/27 2	3.9	3/77 15	5.606 (1.240-25.337)	0.027*
Clarithromycin Missing observations	22.2	6/27 2	6.5	5/77 15	4.114 (1.141-14.835)	0.032*
Diuretics	69.0	20^#^/29	51.1	47^##^/92	2.128 (0.877-5.163)	0.091
- Torasemid	62.1	18/29	32.6	30/92	3.382 (1.420-8.052)	0.005*
- Furosemide	6.9	2/29	3.3	3/92	2.198 (0.349-13.841)	0.592
- Hydrochlorothiazide	6.9	2/29	15.2	14/92	0.413 (0.088-1.934)	0.353
- Potassium-sparing diuretics	6.9	2/29	1.1	1/92	6.741 (0.588-77.227)	0.142
NSAIDs	37.9	11/29	17.4	16/92	2.903 (1.153-7.311)	0.021*
PPI	82.8	24/29	56.5	52/92	3.692 (1.295-10.530)	0.011*
ACE inhibitors/Angiotensin receptor antagonists	75.9	22/29	63.0	58/92	1.842 (0.712-4.764)	0.203
Immunosuppress. Drugs Missing observations	13.8	4/29	19.8	18/91 1	0.649 (0.200-2.101)	0.468
Anticoagulants	10.3	3/29	10.9	10/92	0.946 (0.242-3.699)	1.000
Metformin	3.4	1/29	13.0	12/92	0.238 (0.030-1.915)	0.186
Pre-existing cardiovascular diseases	89.7	26/29	81.5	75/92	1.964 (0.532-7.251)	0.398
Inpatient care Missing observations	82.1	23/28 1	31.9	29/91 1	9.834 (3.397-28.468)	< 0.001*
Consumption of vegetables	41.4	12/29	75.0	69/92	0.235 (0.098-0.565)	0.001*
Consumption of instant food	24.1	7/29	10.9	10/92	2.609 (0.891-7.640)	0.121
Consumption of fruits	20.7	6/29	31.5	29/92	0.567 (0.208-1.541)	0.262
Consumption of pasta	82.8	24/29	81.5	75/92	1.088 (0.363-3.262)	0.880
Consumption of dairy products	48.3	14/29	33.7	31/92	1.837 (0.787-4.284)	0.157
Bottled water	79.3	23/29	80.4	74/92	0.932 (0.331-2.627)	0.895
Non-bottled water	20.7	6/29	17.4	16/92	1.239 (0.435-3.533)	0.688
Juice	48.3	14/29	39.1	36/92	1.452 (0.627-3.363)	0.383
Alcoholic beverages	3.4	1/29	10.9	10/92	0.293 (0.36-2.391)	0.457
Property of companion animals	27.6	8/29	35.9	33/92	0.681 (0.272-1.707)	0.411
Property of birds	13.8	4/29	3.3	3/92	4.747 (0.996-22.618)	0.056

*Significant risk factors (p < 0.05).

*^#^Three C. difficile positive patients consumed more than one diuretic preparation, hence adding up all 4 preparations the total of intaken diuretics is 24.*

*^##^One C. difficile negative patient consumed more than one diuretic preparation, hence adding up all 4 preparations the total of intaken diuretics is 48.*

**TABLE 2:** Results of the multivariate logistic regression model.

Multivariate logistic regression model		
Item	Odds ratio (95% CI)	*p*-value
Prior antibiotic medication	2.715 (0.676-10.903)	0.159
Prior medication clarithromycin	1.530 (0.270-8.666)	0.630
Prior medication meropenem	2.018 (0.345-11.810)	0.435
Torasemid	2.600 (0.780-8.668)	0.120
PPIs	2.506 (0.626-10.028)	0.194
Inpatient care	4.619 (1.300-16.411)	0.018*
Consumption of vegetables	0.240 (0.072-0.796)	0.020*

All factors with p < 0.05 were included in the analysis. Significant factors are marked by*. The item “surgery in previous 12 months” was excluded because the significance was attributed to the extended stay in health care facilities in the previous year.

No significant influence was identified for the holding of companion animals in general (*p* = 0.411). Eight of 29 (27.6%) *C. difficile*-positive patients owned companion animals, including cats, dogs, horses and birds. None of the animals suffered from diarrhea. Two patients allowed recruiting the stool of their animals (horse and chicken). However, in none of the stool specimens *C. difficile* could be identified. However, four of the 29 *C. difficile*-positive patients (13.8%) stated owing birds, remarkably many (*p* = 0.056) in comparison to 3.3% of the *C. difficile*-negative patients (*n* = 3/92).

### Treatment-Based Risk Factors for *Clostridioides (Clostridium) difficile* Infection

*Clostridioides (Clostridium) difficile* Infection was significantly more often a hospital-acquired infection (*p* < 0.001) than diarrhea based on non-communicable diseases or caused by other pathogens, e.g., noroviruses, adenoviruses or *Campylobacter jejuni* (Table 1). 23 of 28 CDI cases (82.1%, 1 missing observation) were hospital-acquired, whereas only 29 out of the 91 *C. difficile*-negative patients (31.9%, 1 missing observation) developed diarrhea inside the health care facility. According to the logistic regression model, the risk for CDI was four- to fivefold higher during a hospital admission (Table 2). However, 17.9% of *C. difficile*-positive patients developed diarrhea outside the health-care facility or within the first 48 h after hospital admission.

Furthermore, whereas only 21 of 92 (22.8%) *C. difficile*-negative patients had experienced surgery in the previous 12 months before this study, this ratio was higher in the group of *C. difficile*-positive individuals (13 out of 29, 44.8%; *p* = 0.022). The majority of CDI patients underwent neurosurgical (*n* = 6/13, 46.2%) or vascular (*n* = 3/13, 23.7%) procedures. Another significant risk factor was the treatment with antibiotics within the previous six months; 82.1% (*n* = 23/28, 1 missing observation) of *C. difficile*-positive patients had been treated with antibiotics in the previous six months prior to the present study. In contrast to that only 48.4% of *C. difficile*-negative patients (*n* = 44/91, 1 missing observation) had received antibiotics before (*p* = 0.002). Especially the patients treated with meropenem or clarithromycin got significantly more often infected with *C. difficile, p* = 0.027 and *p* = 0.032, respectively (Table 1).

Out of all other medications applied (Table 1) the consumption of drugs belonging to three groups demonstrated a significant association with symptomatic CDI; proton pump inhibitors (PPI) were more frequently used by CDI patients (*n* = 24/29, 82.8%) than by those without *C. difficile* (*n* = 52/92; 56.5%, *p* = 0.011). A similar association (*p* = 0.021) was observed with non-steroidal anti-inflammatory drugs (NSAIDs); in contrast to the *C. difficile*-positive patient group (*n* = 11/29; 37.9%) a significant lower percentage of the *C. difficile*-negative patients (*n* = 16/92; 17.4%) had used NSAIDs. Unexpectedly, one specific diuretic preparation, torasemid, also showed a significant association (*p* = 0.005) with CDI; whereas only 30 of 92 (32.6%) *C. difficile*-negative patients used torasemid every day, 18 of 29 (62.1%) *C. difficile*-positive patients took this medication on a daily basis (Table 1). In order to analyze whether diuretics might have a microbiome-modulating effect thereby promoting CDI through inhibiting growth of the intestinal bacterial flora, all diuretics were tested for antimicrobial capacity against fully antimicrobial susceptible *Bacillus subtilis*. This bacterial species is routinely used in laboratories to monitor for antibiotic substances in body fluids. However, none of the tested diuretics had an antibiotic potential (data not shown).

## Discussion

Analyzing a total of 121 patients in a rural area of Germany, we identified *C. difficile* as a likely cause of diarrhea in 24.0% of them. Confirming previous studies, CDI had a significant correlation with hospital-acquired infection in our cohort (Rupnik et al., 2009; Leffler and Lamont, 2015). Our rate of community-acquired CDI is also similar to the results of previous studies which reported a percentage of 20-29% for community-acquired infection (Clohessy et al., 2014; Gupta and Khanna, 2014). Even though the percentage of non-healthcare associated CDI has increased in recent years, the transmission mechanisms largely remain to be disclosed. One reason for this precise lack of knowledge is that the predominantly infected individuals seem not to belong to any high-risk population (Vindigni and Surawicz, 2015).

This study confirmed the association between the occurrence of *C. difficile* and previous antibiotic use (King and Lager, 2011; Badger et al., 2012; Kurti et al., 2015; Leffler and Lamont, 2015). The univariate analysis showed a four- to fivefold higher risk for the acquisition of *C. difficile* after an antibiotic treatment, especially when meropenem and clarithromycin have been used during the previous six months of the beginning of diarrhea (Table 1). Besides a prior antibiotic therapy the consumption of PPIs showed as well a significant association (*p* = 0.011) with the occurrence of CDI. This risk factor has also been described previously in several studies (King and Lager, 2011; Kim et al., 2012; Kurti et al., 2015) and a meta-analysis of more than 10,000 cases of hospital-acquired CDI (Arriola et al., 2016). However, the mechanisms for the association between PPI consumption and CDI are conflicted (Shah et al., 2000; McCarthy, 2012; Trifan et al., 2017). Currently it is merely evident that the concomitant intake of antibiotic preparations increases the risk of getting infected (Kwok et al., 2012). As 24 of 29 (82.8%) *C. difficile*-positive patients consumed both PPIs and antibiotics, our series supports the assumption that a probable association between PPI use and CDI exists, that can be further increased by simultaneous consumption of antibiotics and PPI.

Furthermore the results of our study suggest an association with the intake of NSAID with CDI. Only one other study revealed an association between CDI and the current use of the NSAID diclofenac (Suissa et al., 2012). We observed that 37.9% *C. difficile*-positive participants used NSAIDs during the study interval. However, as all of them also stated the regular intake of PPI, it is more likely that the remarkable effect is mainly caused by PPI.

Moreover a significant correlation between the daily consumption of the loop diuretic torasemid and the occurrence of CDI was observed. Regarding this aspect there are currently only very rare and imprecise published data available. Two older studies reported a significant association between the use of diuretics in general and the occurrence of *C. difficile* without an investigation of the cause-effect relationship (Raveh et al., 2006; Debast et al., 2009). Analyzing this significance, this study examined the antibiotic potential of torasemid, furosemide and hydrochlorothiazide by the use of an agar diffusion test. However, no growth-inhibiting zones were identified in any of the samples indicating that a direct antibiotic potential of the diuretic medication is unlikely the reason for the observed significance. Therefore, additional factors like the indirect effect of torasemid and the extent of cardiac failure on CDI growth should be considered in future studies with patients suffering from CDI. Alternatively, torasemid could stimulate the development of CDI by influencing the bile acid concentration in the human gut in an advantageous way for *C. difficile*. One transporter – the apical sodium-dependent bile acid transporter (ASBT) – is responsible for the re- and absorption of 95% of the bile acids in the human gut (Alrefai and Gill, 2007). So far, only one study about the influence of torasemid on ASBT was found (Zheng et al., 2009). This study disproves an effect on the ASBT, but is only based on an *in vitro* investigation and not on human samples. Our results could also indicate that multimorbid patients, especially with severe cardiac failure for which torasemid is used, suffer in general more frequently from CDI.

The results of our study also suggest an association between the occurrence of CDI and surgical treatment procedures in the year before the study (*p* = 0.022). In line with our data is a multicenter retrospective study that showed increasing duration of postoperative antimicrobial prophylaxis was associated with higher CDI rates (Branch-Elliman et al., 2019). Other retrospective database analyses showed a relatively low incidence of *C. difficile* in surgical patients (Zerey et al., 2007; Jenkins et al., 2010; Guzman et al., 2016). The investigation of Zerey et al. (2007) was the only one evaluating the risk of different visceral surgical procedures on the occurrence of CDI. They described the highest risk for CDI among patients undergoing emergency surgery in general or gastrointestinal surgery (2-3 fold higher risk). In this cohort only one CDI patient underwent surgery of the gastrointestinal tract, the majority (46.2%, *n* = 6/13) had experienced neurosurgical procedures. As this study revealed a health care environment as the most influent risk factor for CDI we state that this is also the most likely reason for the found association between surgical procedures and the occurrence of CDI.

Several studies have discussed asymptomatic carriage of *C. difficile* among animals with differing results (Rodriguez-Palacios et al., 2013). Whereas Schneeberg et al. (2012) claim the asymptomatic carriage in nearly 5.0% of canines and felines living in animal shelters, Weese et al. (2012) state a *C. difficile* prevalence of 10.0% in dogs and 21.0% in cats. Although we were unable to identify *C. difficile* in any of the few animal stool samples provided by their infected owners, a closer look to the owing of animals revealed that diarrheic *C. difficile*-positive patients owned remarkably more birds than diarrheic patients without CDI (*p* = 0.056, *n* = 4/29, 13.8%). Although pet and farm animals have also been confirmed as a source of CDI in a recent review, birds have not yet emerged as risk factor (Crobach et al., 2018).

So far, investigations on food as risk factor have focused mainly on meat products but not on vegetables (Crobach et al., 2018). According to the multivariate logistic regression model applied to our results, a nutrition poor in vegetables was besides inpatient care the only factor showing significance (*p* = 0.020) for the risk of acquiring CDI. With an odds ratio of 0.235, the risk for a CDI was nearly fourfold lower among patients who ate vegetables every day. This suggests a negative influence of an unbalanced nutrition. None of the individuals suffered from any pre-existing illnesses which did not allow eating vegetables. The most frequent preexisting illnesses were of cardiovascular origin. Various studies confirmed the influence of nutrition habits, highlighting the positive effect of fiber and their metabolites on the variability of the human microbiome and its subsequent positive effect against CDI (May et al., 1994; O’Keefe, 2010; Xu and Knight, 2015). This effect was shown to be based on the metabolization of fiber into short chain fatty acids like butyrate which decrease the pH in human gut and thus inhibit the proliferation and the production of toxin A of *C. difficile* (May et al., 1994). The corresponding short chain fatty acids like butyrate are metabolized during the digestion of vegetables. Furthermore, it was proofed in a murine model that diets being rich in saturated fats but lacking vegetables decrease the presence of advantageous microbiota in the gut (Ridaura et al., 2013). The study of May et al. (1994) also underlines the importance of fiber-enriched nutrition for achieving a stable intestinal microbiome for the inhibition of *C. difficile*.

Possible limitations of this study are a recall and misclassification bias by some questions in the survey that were not designed to capture sufficient information or eventually were confounding the results. Exposure factors of the hospital were not within the aims of this study.

## Conclusion

Taken together, in addition to already described risk factors such as inpatient care, antibiotics and PPI, the regular use of torasemid was identified as a potential further risk factor for CDI that should be a subject of future studies. However use of diuretics might only be a surrogate marker for severe heart and renal diseases or in a simplistic view a surrogate marker for higher age or morbidity. The results of our study also revealed a significant impact of nutrition, especially vegetables, on CDI. In particular with regard to the unclear cause of the increasing proportion of community-acquired CDI this aspect awaits further investigations.

## Data Availability Statement

The raw data supporting the conclusions of this article will be made available by the authors, without undue reservation.

## Ethics Statement

The studies involving human participants were reviewed and approved by Ethical Committee of the University Medical Center Göttingen. The patients/participants provided their written informed consent to participate in this study.

## Author Contributions

UG had the initial idea which was developed in a project together with JO and OZ. VL and KG collected the samples and performed the microbiological analyses. VL interviewed the patients and together with all authors interpreted the results. The manuscript was written with contributions of all authors, and all authors read and approved the final version.

## Conflict of Interest

The authors declare that the research was conducted in the absence of any commercial or financial relationships that could be construed as a potential conflict of interest.

## Publisher’s Note

All claims expressed in this article are solely those of the authors and do not necessarily represent those of their affiliated organizations, or those of the publisher, the editors and the reviewers. Any product that may be evaluated in this article, or claim that may be made by its manufacturer, is not guaranteed or endorsed by the publisher.
