# Supplementary material for: Risk Factors of Patients With Diarrhea for Having Clostridioides (Clostridium) difficile Infection
Source: Front Microbiol. 2022 Mar 11;13:840846. doi: 10.3389/fmicb.2022.840846 (PMC8963458; doi:10.3389/fmicb.2022.840846)
Supplement: Supplementary file 1 [file Data_Sheet_1.pdf]

UNIVERSITÄTSMEDIZIN GÖTTINGEN UMG

Universitätsmedizin • Institut f. Med. Mikrobiologie • Kreuzbergring 57, 37075 Göttingen

Institut für Medizinische Mikrobiologie
Nationales Referenzzentrum für Systemische Mykosen
Nationales Konsiliarlabor für Toxoplasmose
Direktor: Prof. Dr. U. Groß

Kreuzbergring 57, 37075 Göttingen
Telefon: 0551 / 39-5806/01
Fax: 0551 / 39-5861
E-Mail: ugross@gwdg.de

A study on microbiological genesis of diarrhea – the role of *Clostridium difficile*

Principal Investigator: Prof. Dr. med. Uwe Groß (contact details see above)

Questionnaire

Dear Madam, dear Sir,

I am from the Institute of Medical Microbiology of the University Medical Center of Göttingen. In our study, we aim to identify risk factors for an illness caused by the bacterium Clostridium difficile, a germ that may induce acute enteritis. The symptoms of this disease can range from mild, self-limiting diarrhea to life-threatening toxic megacolon and peritonitis (Mc Fraland and Stamm, 1986). With the information we collect, we hope to help policy makers to plan better health services.

We are looking for patients with intestinal complaints. It would be of great help to us if you would agree to answer a few questions on your lifestyle and medical history and if you would give us a stool sample for further investigation. Eventually, we would also like to investigate the soil outside of the place you live or a stool sample of your pet, if you own one. All of your answers will be confidential and will not be shared with any others than members of our survey team. After completion of the study, all personalized data will be deleted.

If you agree in this study and do not feel comfortable to answer a specific question, it is no problem, we just go on to the next. You can of course stop the interview at any time. This would have no negative effect on your treatment.

Reference: Mc Farland LV, Stamm WE. Review of Clostridium difficile associated diseases. Am J of Infect Control 1986; 14:99-109.

Date of interview:

Interviewer:

General information

Name

Date of hospital admission: ______________________

Number: ____________________________________

Sex:

- [ ] Female
- [ ] Male

Age: ____________________________________
Place of birth: ____________________________________
Region: ____________________________________
Place of residence: ____________________________________
Religion: ____________________________________

1. Profession:

- [ ] Unemployed
- [ ] Housewife
- [ ] Office worker
- [ ] Farmer
- [ ] Medical sector
- [ ] Trade/industrial worker
- [ ] Pensioner
- [ ] others:

2. Education:

(highest qualification)

- [ ] No education
- [ ] Primary school
- [ ] Secondary school
- [ ] High school graduation
- [ ] Academic degree

Universitätsmedizin Göttingen, Georg-August-Universität Stiftung Öffentlichen Rechts **Vorstand** Prof. Dr. Cornelius Frömmel (Forschung & Lehre, Sprecher des Vorstands) Priv. Doz. Dr. Günther Bergmann (Krankenversorgung) Dipl.-Kffr. (FH) Barbara Schulte (Wirtschaftsführung & Administration) **Sparkasse Göttingen** (260 500 01) Kto: 448

3. Marital status

- [ ] Living in partnership
- [ ] Single

4. Health insurance

- [ ] Yes
- [ ] No

Livestyle information

5. You live together with how many people?

- [ ] 1
- [ ] 2
- [ ] 3-5
- [ ] > 5

6. How many people sleep in one room?

- [ ] 1
- [ ] 2
- [ ] 3-5
- [ ] > 5

7. Do you keep pets?

- [ ] Yes
- [ ] No

Universitätsmedizin Göttingen, Georg-August-Universität Stiftung Öffentlichen Rechts **Vorstand** Prof. Dr. Cornelius Frömmel (Forschung & Lehre, Sprecher des Vorstands) Priv. Doz. Dr. Günther Bergmann (Krankenversorgung) Dipl.-Kffr. (FH) Barbara Schulte (Wirtschaftsführung & Administration) **Sparkasse Göttingen** (260 500 01) Kto: 448

7.1. If yes, which ones (otherwise continue with question 8)?

- ☐ Cats
- ☐ Dogs
- ☐ Goats
- ☐ Sheeps
- ☐ Cows
- ☐ Chickens
- ☐ Ducks
- ☐ Gooses
- ☐ Horses
- ☐ Pigs
- ☐ Others: ________________________________

7.2. Do the animals enter the house?

- ☐ Sleeping room
- ☐ Kitchen
- ☐ Sanitary facilities

8. Where do you get your food from?

Homegrown, own garden

- ☐ Always
- ☐ Mostly
- ☐ Partly
- ☐ Rarely
- ☐ Never

Even producer market

- ☐ Always
- ☐ Mostly
- ☐ Partly
- ☐ Rarely
- ☐ Never

Conventional discounter/Industrially processed food

- ☐ Always
- ☐ Mostly
- ☐ Partly
- ☐ Rarely
- ☐ Never

Universitätsmedizin Göttingen, Georg-August-Universität Stiftung Öffentlichen Rechts **Vorstand** Prof. Dr. Cornelius Frömmel (Forschung & Lehre, Sprecher des Vorstands) Priv. Doz. Dr. Günther Bergmann (Krankenversorgung) Dipl.-Kffr. (FH) Barbara Schulte (Wirtschaftsführung & Administration) **Sparkasse Göttingen** (260 500 01) Kto: 448

9. Do you wash/clean vegetables and fruits before eating?

- ☐ Always
- ☐ Mostly
- ☐ Partly
- ☐ Rarely
- ☐ Never

10. Which three of the following foods do you eat mostly (at least one portion every day)?

- ☐ Fruits
- ☐ Vegetables
- ☐ Fish
- ☐ Meat
- ☐ Instant food (including canned food, dried soups)
- ☐ Carbohydrates (corn, rice, pasta, bread)
- ☐ Milk products

11. Are you

- ☐ Meat eater
- ☐ Vegetarian
- ☐ Vegan
- ☐ Others

12. How much liquid do you consume per day?

- ☐ < 0,5 l
- ☐ 0,5 – 1,0 l
- ☐ 1,0 – 1,5 l
- ☐ 1,5 – 2,0 l
- ☐ > 2,0 l

13. What is your main water source?

- ☐ Tap water
- ☐ Bottled water

14. What else do you drink (multiple answers possible)?

- ☐ Raw milk
- ☐ Pasteurised milk
- ☐ Juices
- ☐ Softdrinks (coke etc.)
- ☐ Alcoholic beverages

Universitätsmedizin Göttingen, Georg-August-Universität Stiftung Öffentlichen Rechts **Vorstand** Prof. Dr. Cornelius Frömmel (Forschung & Lehre, Sprecher des Vorstands) Priv. Doz. Dr. Günther Bergmann (Krankenversorgung) Dipl.-Kffr. (FH) Barbara Schulte (Wirtschaftsführung & Administration) **Sparkasse Göttingen** (260 500 01) Kto: 448

☐

15. How many meals do you eat per day?

☐ 1
☐ 2
☐ At least 3

16. Does your family have enough to eat?

☐ Yes
☐ No

Medical information

17. Height(cm)

____

18. Weight (kg)

____

19. Pre-existing illnesses, infections, surgeries in the last 12 months

☐ Tuberculosis
☐ Hepatitis A
☐ Hepatitis B
☐ HIV/AIDS
☐ Giaria, Lamblia
☐ Diabetes
☐ Thyroid diseases
☐ Cardiac diseases
☐ Renal failure
☐
☐
☐

20. Recent complaints/ admission diagnosis

____

____

____

Universitätsmedizin Göttingen, Georg-August-Universität Stiftung Öffentlichen Rechts **Vorstand** Prof. Dr. Cornelius Frömmel (Forschung & Lehre, Sprecher des Vorstands) Priv. Doz. Dr. Günther Bergmann (Krankenversorgung) Dipl.-Kffr. (FH) Barbara Schulte (Wirtschaftsführung & Administration) **Sparkasse Göttingen** (260 500 01) Kto: 448

Information about the gastrointestinal status:

21. Last bowel movement ________________ days

22. Do you feel abdominal pain

0	1	2	3	4	5	6	7	8	9	10

0: no pain
10: strongest pain

23. Do you suffer from flatulence?

☐ Yes
☐ No

24. How is your diarrhea?

☐ Acute ☐ Chronic

25. For how long did you have diarrhea?

___

26. How many times do you have bowel movement per day?

___

27. How is the consistency of your stool?

☐ Pasty
☐ Liquid
☐ Slimy
☐ Foamy
☐ Solid
☐ Brown
☐ Other colour than brown
☐ Bloody

Other characteristics

___

Universitätsmedizin Göttingen, Georg-August-Universität Stiftung Öffentlichen Rechts **Vorstand** Prof. Dr. Cornelius Frömmel (Forschung & Lehre, Sprecher des Vorstands) Priv. Doz. Dr. Günther Bergmann (Krankenversorgung) Dipl.-Kffr. (FH) Barbara Schulte (Wirtschaftsführung & Administration) **Sparkasse Göttingen** (260 500 01) Kto: 448

Other gastrointestinal complaints

28. Do you feel pain during defecation?

0	1	2	3	4	5	6	7	8	9	10

0: no pain
10: strongest pain

29. Did anyone of your family have similar symptoms?

- [ ] Yes
- [ ] No

30. How were/are you treated?

- [ ] No treatment
- [ ] Self-treatment
- [ ] Medical treatment
- [ ] Others
- [ ]
- [ ]

32. Recent medications

Indication	Name	Dosis	Frequency	For how long

33. Were you treated with antibiotics in the last 6 months?

- [ ] Yes — Name of the preparation/s ____________________
- [ ] No

34. Intake of supplements/ herbal medicines?

Indication	Name	Dosis	Frequency	For how long

Universitätsmedizin Göttingen, Georg-August-Universität Stiftung Öffentlichen Rechts **Vorstand** Prof. Dr. Cornelius Frömmel (Forschung & Lehre, Sprecher des Vorstands) Priv. Doz. Dr. Günther Bergmann (Krankenversorgung) Dipl.-Kffr. (FH) Barbara Schulte (Wirtschaftsführung & Administration) **Sparkasse Göttingen** (260 500 01) Kto: 448

Thank you very much for your participation!

Universitätsmedizin Göttingen, Georg-August-Universität Stiftung Öffentlichen Rechts **Vorstand** Prof. Dr. Cornelius Frömmel (Forschung & Lehre, Sprecher des Vorstands) Priv. Doz. Dr. Günther Bergmann (Krankenversorgung) Dipl.-Kffr. (FH) Barbara Schulte (Wirtschaftsführung & Administration) **Sparkasse Göttingen** (260 500 01) Kto: 448
